# Growth and survival of the superorganism: Ant colony macronutrient intake and investment

**DOI:** 10.1002/ece3.6520

**Published:** 2020-07-07

**Authors:** Yeisson Gutiérrez, Tung Phung, Harald Mumma, Arden Ambrose‐Winters, Christoph Scherber, Chris R. Smith

**Affiliations:** ^1^ Institute of Landscape Ecology University of Münster Münster Germany; ^2^ Department of Biology Earlham College Richmond IN USA

**Keywords:** nutritional ecology, resource imbalance, social insect, stable isotopes, stoichiometry

## Abstract

In this study, we used two common ant species (*Lasius niger* and *Lasius neoniger*) to assay how they translate variation in the diet (both in composition and frequency) into growth. We measured colony development for over 8 months and measured several phenotypic traits of the worker caste, and examined whether forager preference corresponded with diet quality. Optimal colony growth was a balance between survival and growth, and each of these was maximized with different nutrient regimes. Interestingly, forager preference was not totally aligned with the diet that maximized colony growth. Our results highlight that: (a) organism and superorganism size are controlled by the same nutrients, and this may reflect a common molecular basis for size across life's organizational levels, (b) there are nutrient trade‐offs that are associated with life‐history trade‐offs, likely leading to selection for a balanced diet, and (c) the connection between the preference of foragers for different nutrients and how nutrient combinations affect colony success and demographics are complex and only beginning to be understood.

## Graphical abstract

Nutrients can limit how organisms allocate to different necessary life processes (life‐history allocations). We show that ant colonies and the individual ants making up the colonies show similar trade‐offs with regard to the amount of protein and carbohydrate that is available to them, and that both individuals and colonies have the same optimal ratios of these necessary macronutrients.

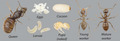

## 1 INTRODUCTION

Nutrition is an upstream organizer of most organismal processes, and thus dealing with variation in the availability of nutrients is among the most consequential challenges, and selective agents, faced by all organisms (Stearns, 1992; Zera & Harshman, 2001). While many nutrients are necessary for maintenance and growth, the majority consumed and used are macronutrients ingested in high quantity, such as proteins, carbohydrates, and fats (Raubenheimer, Simpson, & Mayntz, 2009). The molecular mechanisms that translate incoming macronutrients into maintenance, new tissue, or storage are well understood, and tend to be highly conserved (Conlon & Raff, 1999).

Superorganisms, like eusocial insects, are groups of individuals of the same species operating in a synergistic way (Wilson & Sober, 1989). Superorganisms are characterized by the reproductive division of labor, such that some individuals do not reproduce (somatic germ line division) (Hölldobler & Wilson, 2009). The nutritional challenges of any cell or organism are also faced by superorganisms, but are compounded by the additional level of complexity (cooperating organisms on top of cooperating cells, etc.)—the labor that is divided among cells in “unitary” organisms is divided among individuals in the superorganism (Crumière, Stephenson, Nagel, & Shik, 2020; Csata & Dussutour, 2019). How, then, do nutritional challenges scale from organism to superorganism? Do organism and superorganism have different nutritional optima and/or face the same nutrition‐mediated trade‐offs? If individual and superorganism optima are not aligned, then processes occurring at the individual level may limit those at the superorganismal level.

Social insect colonies undergo a coordinated development that is responsive to the environment and also lineage/species specific, termed “sociogenesis” (Wilson, 1985). “Sociometry” is a general term applied to the metrics associated with superorganisms, such as colony size, individual size (and size distributions), the numbers and ratios of different castes, among others. As a general pattern in studies of sociometry and sociogenesis, worker size increases with the number of workers in the colony (Smith & Tschinkel, 2006; Tschinkel, 1987, 1993, 1998). The patterns observed in studies on the sociometry and sociogenesis of colonies are linked to mechanistic studies on the effects of nutrition on colony properties. The conversion of available nutrients into new ants is governed by a complex set of internal feedbacks within colonies (Cassill & Tschinkel, 1995; Dussutour & Simpson, 2009), as is the manner in which nutrients relate to the processes that govern ant development and phenotypic plasticity (both within and among castes). In general, manipulative studies have shown that increases in bulk nutrients increase the number and size of individuals produced in a colony (Aron, Keller, & Passera, 2001; Smith, 2007; Wills et al., 2015), in agreement with the hypothesis that larger colonies produce more and larger workers because they have a greater access to nutrients. Specific nutrients are also known to affect allocation within colonies, for example, increased protein tends to increase individual size (Aron et al., 2001; Bono & Heithaus, 2002; Goetsch, 1937; Hunt & Nalepa, 1994; Passera, 1974; Schmidt, Hunt, & Smith, 2012; Smith, Anderson, Tillberg, Gadau, & Suarez, 2008; Smith & Suarez, 2010; Smith, 1942); access to carbohydrates has been shown to have a similar effect (Wills et al., 2015), but the mechanism is likely an increase in worker longevity rather than the production of new workers (Dussutour, Poissonnier, Buhl, & Simpson, 2016). Worker number and size can also trade‐off (Wills et al., 2015), as can the production of different castes (Aron et al., 2001; Smith, 2007). However, excessive amounts of protein in diets can lead to high worker mortality (Arganda et al., 2017; Dussutour & Simpson, 2009, 2012; Poissonnier, Simpson, & Dussutour, 2014). On the other hand, a high carbohydrate diet can lead to increased worker fat mass (Grover, Kay, Monson, Marsh, & Holway, 2007) and increased worker longevity (Dussutour et al., 2016). It is currently unclear how protein and carbohydrates interact to affect metrics of sociometry and sociogenesis.

**FIGURE 1:**
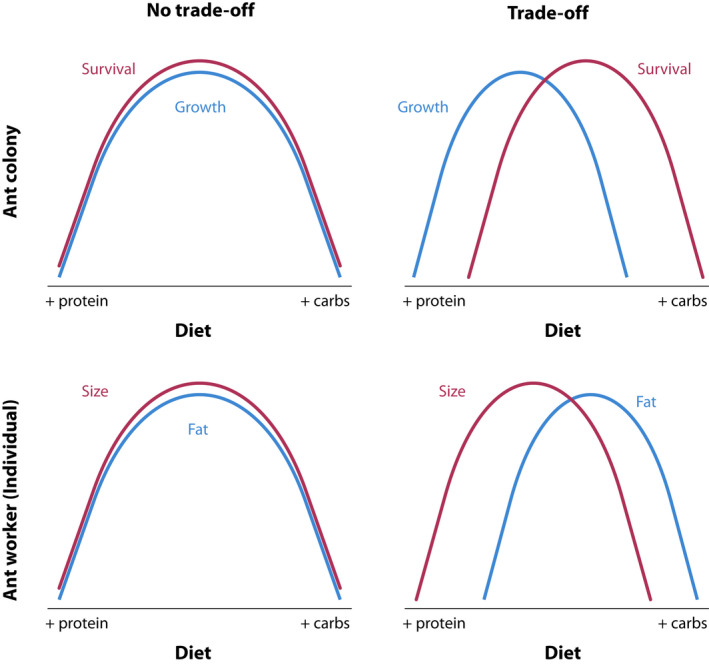
A conceptual schematic of the relationship between nutrient availability/use and trait value for both social colonies (top) and individuals within colonies (bottom). The plots on the left show overlapping optima for different social or individual traits (no trade‐off) and the plots on the right show different optima and thus trade‐offs (intersecting lines)

In the present study, we employed a nutritional geometry framework (Simpson & Raubenheimer, 1995) that allowed for the manipulation of both the ratio and amounts of protein and carbohydrate available to colonies. Combining the prior work on sociogenesis with the shorter‐term manipulative studies, we hypothesized that worker size and colony size would both increase with increasing availability of protein (relative to carbohydrates) in the diet. An important alternative hypothesis is that worker and colony development are limited by different nutrients (i.e., worker and colony optima differ). For example, worker size may indeed be protein limited, but colonies may be more carbohydrate limited due to the metabolic demands of the standing crop of adult workers (see Wills et al., 2015). This alternative hypothesis is premised on there being a nutritionally mediated life‐history trade‐off between growth (new tissue) and maintenance (current tissue, Figure 1).

In addition to the above primary hypothesis, our study addressed two questions related to the mechanisms driving the main result that colony growth and survival are maximized by different macronutrients (Figure 1): (a) how do colonies distribute key macronutrients within the nest, and (b) do colonies have preferences for macronutrients that align with what maximizes colony growth? We examined the flow of nutrients through two types of individual, larva and worker, using a stable isotope tracer experiment. We hypothesized that protein would be more rapidly and completely transferred to larvae (because they are growing) while carbohydrates would persist longer in workers (to maintain worker activity levels). That is, the growth–survival trade‐off might largely be driven by the competing needs of workers and larvae.

In order to examine colony preference for macronutrient ratios, we offered the same diets used in the above experiments to natural and laboratory colonies. Ant foragers have previously been shown to be highly tuned to the nutrient needs of colonies, solving highly multidimensional nutrient challenges (Csata et al., 2020). Therefore, we hypothesized that colonies would prefer diets that maximized laboratory colony growth despite possible competing needs of different castes within the nest. An alternative hypothesis is that worker preference is not aligned with colony growth (under laboratory conditions), but instead is more tuned to the adult worker optimum of high carbohydrates. Other alternative hypotheses include that colonies respond based on experience or environmental variation, among other factors.

## 2 MATERIALS AND METHODS

### 2.1 Study species

**FIGURE 2:**
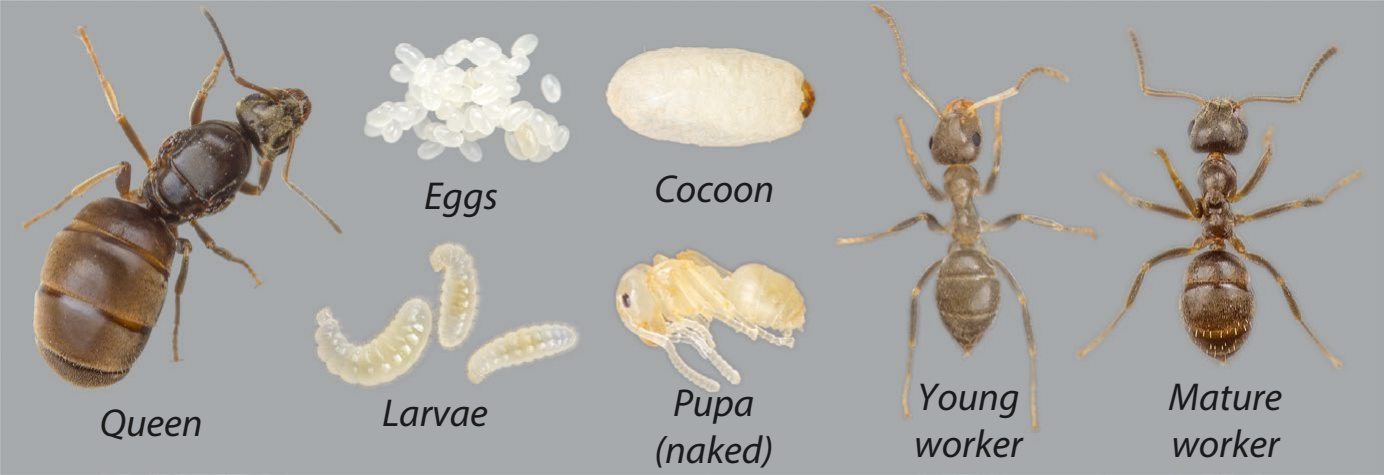
Castes and developmental stages present in a *Lasius niger* colony. Pictures were taken using a Canon 550D camera with a 100 mm macro‐lens and extension rings attached. Figures are not at scale

The experimental procedure was carried out simultaneously for two ant species: *Lasius niger* (L., 1758) (Figure 2) commonly termed as the black garden ant, widespread in Europe and northern America (Klotz, 2008); and *Lasius neoniger* Emery, 1893 known as the turfgrass ant, distributed in northern and Midwestern North America (Wilson, 1955). Both species are highly abundant in urban/suburban lawns and gardens and have similar diets. Natural diets are hemipteran honeydew and living and dead insects (Klotz, 2008; Wilson, 1955).

*Lasius niger* (Figure 2) queens were collected in Münster (NRW, Germany) during a single nuptial flight on July 5, 2017. *Lasius neoniger* queens were collected in Richmond (IN, USA) during two nuptial flights on June 30 and July 12, 2017. Queens were housed in 18 × 150 mm test tubes half‐filled with water and plugged with cotton. Most females initiated egg laying after the first week. No food was provided until the emergence of the first workers' cohort. Once workers were present, colonies were housed in 500 ml plastic boxes containing cotton‐stoppered test tubes with water. Box walls were covered with fluon (polytetrafluoroethylene) to prevent escape. Colonies were housed in incubators at 25°C for *L. niger* and to 30°C for *L. neoniger*. Colonies were overwintered at 10°C (*L. niger*) and 15°C (*L. neoniger*) for 2 and 1 months, respectively, between January and February 2018. An acclimatization phase of 15 days prior and post overwintering period was allowed for gradual temperature transition. The higher temperature used in *L. neoniger* was due to concurrent experiments carried out with multiple, ecologically different, species. Differences in rearing and overwintering temperatures were due to differences in laboratory constraints.

### 2.2 Colony growth

We conducted a pilot study on *L. neoniger* that followed 24 colonies monitored over 6 months. These colonies were randomly assigned to diets differing in the protein:carbohydrate (P:C) ratio of their diet. Data collected from this preliminary experiment allowed us to determine that colony growth (the number of workers) increased near linearly with increasing parts of carbohydrates (1:1 < 1:2 < 1:3, P:C) (Figure S1). These results informed our choice of diets for our main experiment, and additionally, documented that a weekly provision of ~125 μl of artificial diet (see below) was sufficient to maintain colony growth and survival. In this experiment, we aimed to find both the maximum P:C for colony growth and a level so low in protein (relative to carbohydrates) that colony growth declined. Therefore, we started at a diet that we knew was below the optimum for growth, 1:2, and increased carbohydrates relative to protein by eightfold to a level that we hypothesized would be beyond the optimum.

**FIGURE 3:**
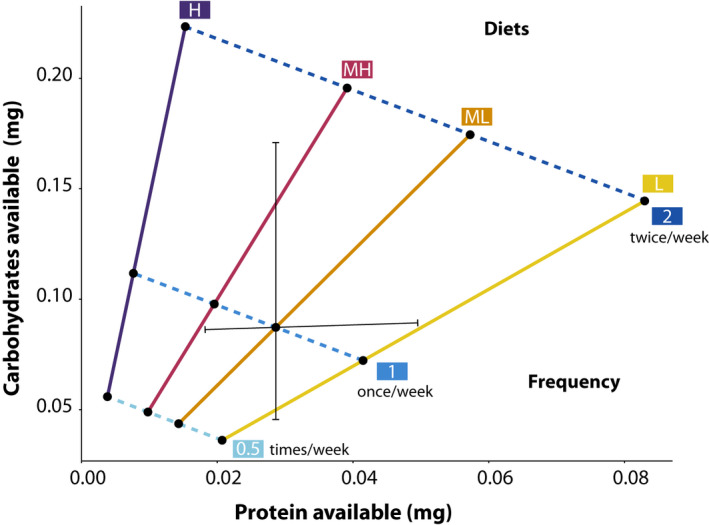
Incipient ant colonies were allocated to twelve experimental treatments (black dots) resulting from the combination of four diets and three feeding frequencies. Values represent weekly available nutrients. Black lines represent the range of protein and carbohydrates used for diet preference assay in field colonies of *Lasius neoniger*

Colonies in the present study were randomly assigned to treatments after the first census (approximately 1 month of growth). The treatments were feeding frequency and diet composition (P:C) (Figure 3). Feeding frequency had three levels: a 125 μl aliquot every other week (0.5), every week (1), or twice per week (2). The P:C had four levels representing a linear change in P:C from approximately 1:2 up to 1:16 with increments of rough doubling (see Figure 3 for details). The exact ratio used differed slightly between species, as follows (*L. niger*/*L. neoniger*): 1:2/1:1.7; 1:4/1:3, 1:8/1:5, 1:16/1:14.5 (see Table S1 for details). For simplicity, the ratios for *L. niger* are those used in figure and table labels. The difference in diets between species was an unintentional artifact of differing laboratory protocols—though the diets of both species are roughly linear and represent similar increments. The starting number of colonies was 96 for *L. neoniger* (eight colonies per treatment combination) and 86 for *L. niger* (7–8 colonies per combination).

Portions were 125 μl (127.33 ± 0.38 mg, mean ± *SD*) of isocaloric chemically defined diets prepared using casein, whey protein, egg powder, and sucrose in agar solution (Dussutour & Simpson, 2008). Insect vitamins (Vandersant, MP Biomedicals) and a preservative (Methylparaben, Dephyte) were also added equally to the diets. For details on the diet constitution and recipes, see Table S1. Food leftovers were removed after 3 days, and new batches of food were prepared every 3 weeks. The development of the colonies was monitored over 8 monthly censuses (disregarding the months spent overwintering) by assessing the worker number. Every colony census was done in a noninvasive way by visually inspecting the test tubes under a stereomicroscope (SZX7, Olympus) at 40× magnification. Colonies were considered “dead” only if the queen died.

Data were analyzed to answer three related questions: (a) Did colony survival vary depending on treatments? (b) Did the dynamics of growth vary with treatments? and (c) Did overall growth vary with treatments?

G‐tests (function “G.test”) were used to assess differences in colony survival as related to treatments using the library RVAideMemoire (Hervé, 2020). The “G.multicomp” function was used for post hoc comparisons—*p*‐values were corrected for false discovery rate (FDR).

A linear mixed‐effects model for each species, function “lme” from library nlme (Pinheiro, Bates, DebRoy, Sarkar, & R Core Team, 2020), was used to assess growth dynamics. Growth was approximated as the number of workers in each monthly census, excluding colonies that died. Feeding frequency, diet P:C, and census number (the latter as a third‐order polynomial identified after initial visual inspection of the data) were the fixed effects (factorial), and colony ID was expressed as a random factor (96 colonies for *L. neoniger* and 86 for *L. niger*). Additionally, census number was added as a first‐order autocorrelation structure (corAR1) grouped by colonies.

To assess the effects of treatments on overall growth, we used a two‐way analysis of variance with the frequency of feeding and diet P:C as fixed effects and the difference in numbers of workers between the last and second census (i.e., the first census that had experienced the treatments) as the response variable.

### 2.3 Worker phenotype

Several different worker traits were measured: head width, dry mass, lean mass, and lipids content. These characteristics, individually and in aggregate, are typically responsive to nutrition and change with colony‐level development and health (Smith & Tschinkel, 2009; Tschinkel, 1993). Head width of young workers (recognized by their lighter coloration, Figure 2) was used as a proxy of body size (Schwander, Rosset, & Chapuisat, 2005). Head width images were taken at 56× magnification using a stereomicroscope (SZX7, Olympus) with an attached camera (Retiga 2000R, Q‐Imagine) and measured at the maximal distance across the eyes using iSolutions‐Lite software (iMT Technology). The number of ants measured per colony varied due to availability (8 ± 2.6, mean ± *SD*). Lipid extractions were done following the protocol of protocol of Smith & Tschinkel, (2009). In brief, samples were first dried for >48 hr at 60°C and weighed. Subsequently, we repeatedly washed the samples with diethyl ether (Merck KGaA) in a Soxhlet extractor during 24 hr. Finally, the ants were dried again after for >48 hr at 60°C and weighed. This process yielded three measures, namely dry mass, lean mass, and the proportion of dry mass that was lipids (i.e., the portion extracted, calculated as (dry mass–lean mass)/dry mass). Due to the low mass of *Lasius* workers, we extracted a sample of ants, *en masse*, for each colony. The number of ants per colony was 8.8 ± 1.8 (mean ± *SD*). Both dry mass and lean mass were normalized as the mass of the sample divided by the number of ants in the sample. Linear models (“lm” function) were used to analyze dry mass, lean mass, and lipid content; head width was analyzed using generalized linear models (“glm” function) with Gamma distribution.

### 2.4 Stable isotopes pulse experiment

In order to examine the flow of nutrients into a colony, we used a stable isotope pulse. Diets enriched with the heavy stable isotopes ^15^N and ^13^C (when abbreviating carbon with C, we use the superscript for atomic mass in order to differentiate from C for carbohydrates) were fed once, and the flow of the isotopes to different castes was assayed over time. After the final census of *L. neoniger*, a subset of eight colonies in the ML treatment were randomly assigned to either of two treatment groups, those receiving the heavy isotope enriched diet (Table S1) or those receiving an unenriched (but otherwise identical) control diet. Five of the eight colonies were provided with the enriched diet, and the remaining three were fed the unenriched diet in order to control for background isotopic levels. The 1:4 diet used in the previous experiment was modified so that isotopically labeled materials could be easily integrated. The basic methods of Shik, Rytter, Arnan, and Michelsen (2018) were followed. A small quantity of ^15^N labeled or unlabeled ammonium nitrate (labeled: Ammonium nitrate‐15N, EB0056; unlabeled: Ammonium Nitrate SHBJ1050, Sigma‐Aldrich) and a small quantity of ^13^C labeled or unlabeled glucose (labeled: D‐Glucose‐1‐13C, MBBC0227V; unlabeled: D‐(+)‐Glucose, SLBJ0583V, Sigma‐Aldrich) were added so that the P:C ratio of the diet was not changed. The supplemental ammonium nitrate amounted to 0.9% (by dry mass) of the protein sources in the diet, and the supplemental glucose was approximately 4% of the carbohydrate sources in the diet. Colonies were offered 125 μl of the diet for 24 hr. After this period, the food was removed and a random sample of approximately five workers and five (mostly last instar) larvae was withdrawn, frozen for 24 hr, and then stored in a drying oven at 60°C (following the methods of Smith & Tillberg, 2009). Colonies were then sampled again after 96 hr following the same procedure. Dried samples were homogenized in sterile aluminum capsules, and elemental analysis was carried out at the University of California at Davis Stable Isotope Facility using a PDZ Europa ANCA‐GSL elemental analyser interfaced to a PDZ Europa 20‐20 isotope ratio mass spectrometer (Sercon Ltd.). The atomic per cent enrichment was calculated as the atomic per cent of the heavy isotope (relative to the lighter isotope) divided by the same quantity for the same sampling time and caste in the unlabeled colonies. The individuals in colonies fed enriched diets, overall, had 9% more ^13^C and 71% more ^15^N compared to individuals in colonies fed unlabeled diets. Each element was analyzed separately using the atomic per cent excess as the response variable. Time (24 vs. 96 hr) and developmental caste (worker vs. larva) were the categorical predictors. Due to heterogeneity of variances, these data were analyzed using a Kruskal–Wallis test followed by a Dunn test (“dunnTest” function in the FSA library; Ogle, 2020) for pairwise differences using the Benjamini–Hochberg method.

### 2.5 Diet preference under field and laboratory conditions

Field trials were conducted between May and June 2018 in grassy lawns for both naturally occurring colonies of *L. niger* (*N* = 23) at the Botanical Garden of Münster (51°57′55.9″N 7°36′22.9″E) and for *L. neoniger* (*N* = 30) on the campus of Earlham College in Richmond, Indiana (39°49′15.2″N 84°54′47.0″W). A 7.5 cm microscope slide with four 125 μl portions of every P:C ratio (1:2, 1:4, 1:8 and 1:16) in random order was placed near an active nest entrance. After placement, the number of ants at each food portion was counted every 5 min during 40 min. If other ant species were seen at any food portion at any location, then that time point and all subsequent time points at that location were not included in the analysis. We assumed that all food portions had an equal probability of discovery. Baited nest entrances were at least 1.5 m apart, and each was used only once. Previous studies have shown that *Lasius* colonies usually have one or few open nest entrances (Traniello & Levings, 1986), and the foraging activity of the workers is concentrated between 10 and 20 cm from the nest entrance (Traniello, 1983).

After food preference for the four P:C levels had been established, we performed an additional diet choice test with the species *L. neoniger* only. The diet was modified to ascertain whether P:C preference was driven by P content and/or C content. Levels of P were varied (holding C constant), and then separately, levels of C were varied (holding P constant). We used the midpoint for these diets to be the most preferred 1:4 diet (see Figure 3) and then halved and doubled either P or C. We then presented three sets of diets to field and laboratory colonies (as above)—all paired diets had either constant C or constant P (i.e., they were either on the vertical or the horizontal black line in Figure 3).

Diet preference data collected from field colonies were square‐root transformed, in order to homogenize variances, and analyzed using a mixed‐effect model to take into account the nonindependence of repeated counts at each colony. The model included the “bait” type (i.e., the diet P:C) as a fixed factor, and colony ID as a random factor (18 colonies for *L. neoniger* and 23 for *L. niger*). A first‐order autocorrelation structure (corAR1) was specified as “Time | colony ID/ bait type”. As above, the “lme” function from the nlme library (Pinheiro et al., 2020) was used to analyze data.

Food preference trials were also conducted on two laboratory‐reared *L. neoniger* colonies (c.a. 2 years old with over one hundred workers). Food portions were placed in foraging arenas attached to colony containers, and then, foraging was filmed using an Apple iPhone 4 with the Lapse‐It (2017 free version, http://www.lapseit.com/) application for time‐lapse. Pictures were taken every 5 min for at least 3.5 hr, and all ants at each bait were counted for every time period. Laboratory data were not statistically analyzed due to low sample size. Both colonies showed the same preference to the four diet ratios, but only one colony of the pair responded with high recruitment to the variable P and C diets (Figure S2).

### 2.6 General data analysis

All analysis and modeling were conducted for each species separately as the aim of this research was not to compare the species but to study the effects of the treatments simultaneously for both of them. All data analysis was done in R version 3.6.2 (R Core Team, 2019). Type II analysis of variance tables based on 1‐*df* chi‐square tests were used to assess the significance of terms in all models (e.g., lm, glm, and lme) using the “ANOVA” function in the “car” library (Fox & Weisberg, 2018); these tests are marginal, that is, each term is tested in presence of all other terms in the model and tests are not order‐dependent. Data were summarized using libraries plyr (Wickham, 2011), dplyr (Wickham, Francois, Henry, & Muller, 2020), and Rmisc (Hope, 2013). Figures were made after the means and confidence intervals calculated from the raw data using ggplot2 (Wickham, 2016) with RColorBrewer (Neuwirth, 2014).

## 3 RESULTS

### 3.1 Colony survival and growth

**FIGURE 4:**
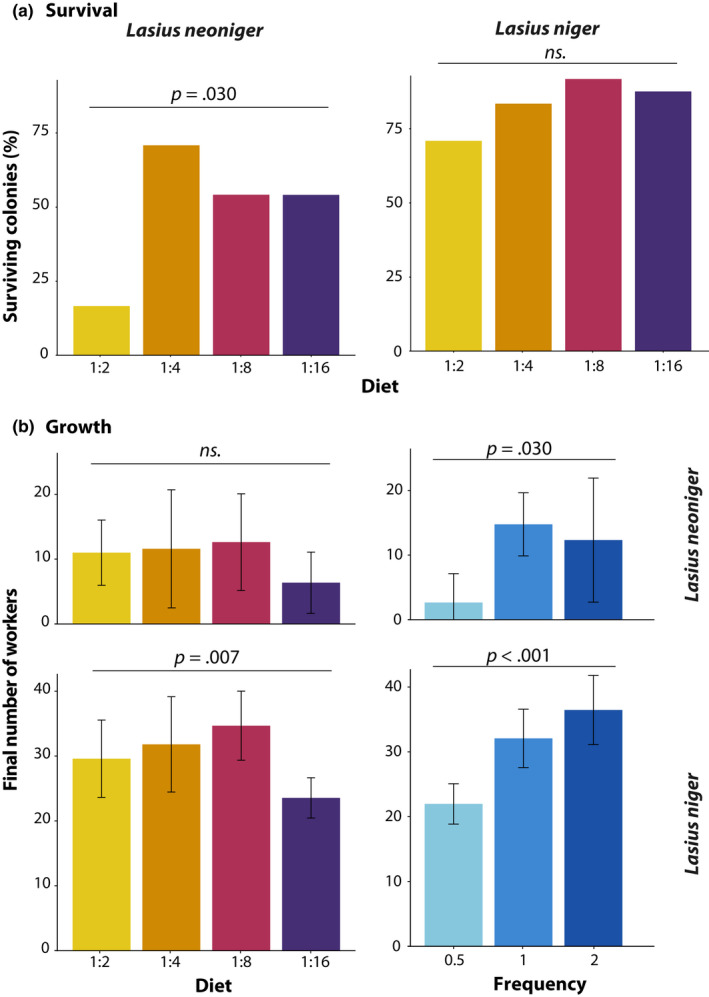
Colony growth and survival. (a) Survival of colonies in *Lasius neoniger* was affected by diet composition (P:C) but not by feeding frequency. In (b) Colony growth was affected by both diet composition and diet frequency in both species, but these factors did not interact—the 1:16 diet had lower growth than others, as did the lowest feeding frequency. Statistical results for colony growth are available in Table S2

**FIGURE 5:**
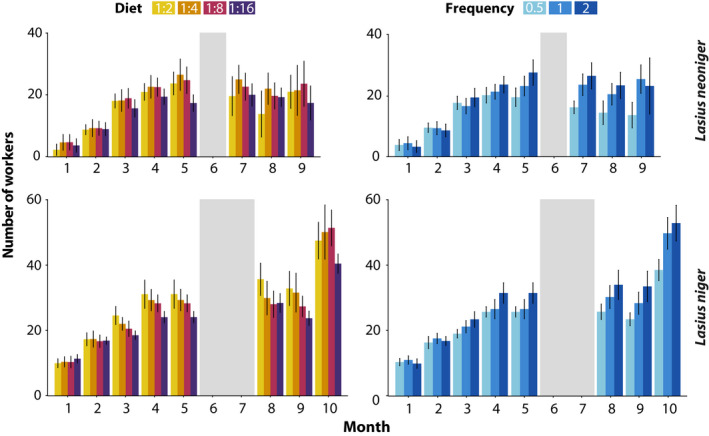
Colony development was affected by diet composition (Protein:Carbohydrates) and feeding frequency, and the dynamics changed over time. Gray areas represent the overwintering perod for both *Lasius neoniger* (1 month) and *L. niger* (2 months). Variation expressed as confidence intervals. Statistical results available in Table 1

The first census of colony growth was performed 1 month after collecting the queens of both species, and the eighth census, nine (for *L. neoniger*) and 10 (for *L. niger*) months later (months spent overwintered were not included in the analysis). Colony survival was affected only by diet treatment in *L. neoniger* (G = 9.19, *df* = 3, *p* = .03, Figure 4a), but neither species showed an effect of feeding frequency on survival. Only the diet low in carbohydrates (1:2) showed decreased survival relative to all other treatments (*p* < .05 in all comparisons after FDR correction). For both species, each fixed effect (diet P:C, feeding frequency, and time) in the mixed‐effects model was statistically significant, as were the interactions Time × Diet and Time × Frequency (Figure 5, Table 1). The latter two‐way significant interactions suggest that the dynamics of diet P:C and feeding frequency changed with time, some of which were likely due to differences becoming more pronounced over time. Additionally, species‐specific effects on growth were apparent from the nonrecovery of *L. neoniger* colonies postwinter, especially due to differential survival (above).

**TABLE 1:** Mixed‐effect model results for colony development

	*df*	*Lasius neoniger*		*Lasius niger*	
		*χ* ^2^	*p*	*χ* ^2^	*p*
Time^3^	3	564.1778	<.001	143.4620	<.001
Diet (P:C)	3	9.8812	.0196	14.6190	.0022
Frequency	2	11.9138	.0026	29.7650	<.001
Time^3^ * Diet	9	24.4371	.0037	44.2110	<.001
Time^3^ * Frequency	6	57.3400	<.001	54.6000	<.001
Diet * Frequency	6	7.3848	.2867	10.0910	.1209
Time^3^ * Diet * Frequency	18	24.2979	.1455	20.4200	.3097

Abbrevitaion: *df*, degrees of freedom.

In the analysis of overall growth, diet P:C and feeding frequency were statistically significant for both species, but there were no significant interactions (Figure 4b, Table S3). The high carbohydrate diet (1:16) produced the lowest growth, differing from the low carbohydrate (L, *p* < .05) and medium‐high carbohydrate (MH, *p* < .005) diets in post hoc comparisons. The frequency that colonies were fed was highly significant, with only colonies fed every other week suffering reduced growth relative to the other two treatment levels (*p* < .001 in both post hoc comparisons). Colonies of *L. niger* grew larger than colonies of *L. neoniger*, seemingly owing to differences in overwintering and recovery from winter.

### 3.2 Worker phenotypes

**FIGURE 6:**
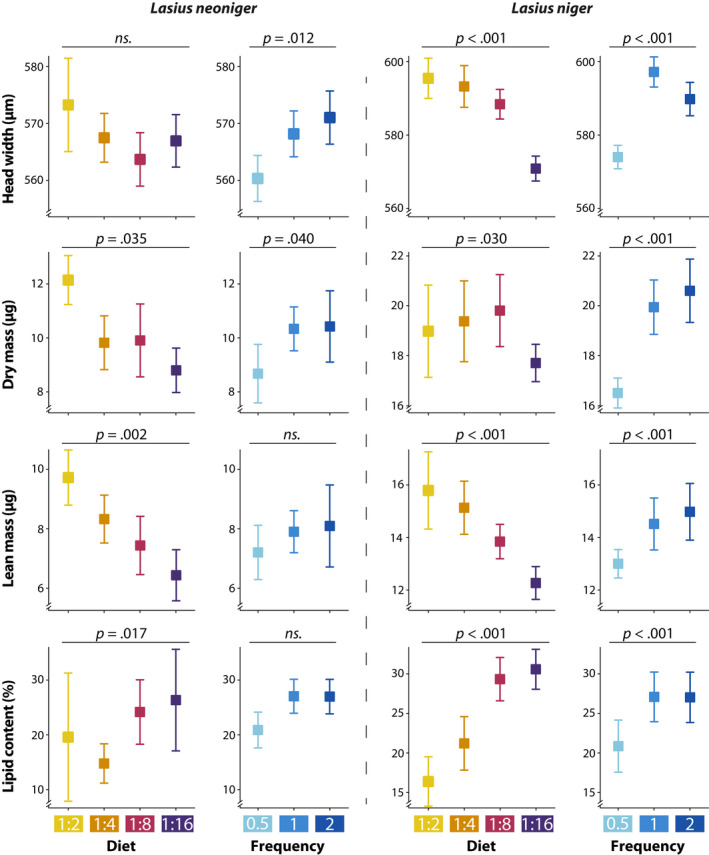
Workers phenotypic traits were responsive to diet P:C and feeding frequency. Higher dietary protein led to bigger workers and less lipid storage, and more frequent feeding was associated with bigger workers and increased fat storage. Variation expressed as confidence intervals. Statistical results are available in Table S4

Differences among treatments in worker phenotypes are summarized in Figure 6 for both species, and full results are given in Table S4. All measured traits were significantly affected by diet P:C and feeding frequency separately in *L. niger*, but only head width exhibited a significant diet P:C × Frequency interaction. In *L. neoniger*, worker lean mass and lipid content were affected by diet P:C only, whereas head width was affected by feeding frequency only. *Lasius neoniger* worker dry mass was the only trait affected by both diet P:C and feeding frequency, and no interactions were significant for all the measured traits of this particular species.

Overall, increasing protein content in diet increased head width, lean and dry mass (Figure 6). But as noted above, the effect was the opposite for worker lipid content. Although *L. neoniger* head width was not affected by diet P:C, the directional difference in means is consistent with the results obtained for lean and dry mass (i.e., more proteinaceous diets correspond to larger workers).

In addition, while feeding frequency only had a statistically significant effect on *L. neoniger* worker dry mass (both higher frequency treatments were marginally different than the lower frequency, *p* = .06 and *p* = .08), the direction of the effect was consistent across all dependent variables assessed for both species (Figure 6)—more frequent feedings (i.e., greater food availability) led to larger and fatter individuals.

### 3.3 Stable isotope pulse experiment

**FIGURE 7:**
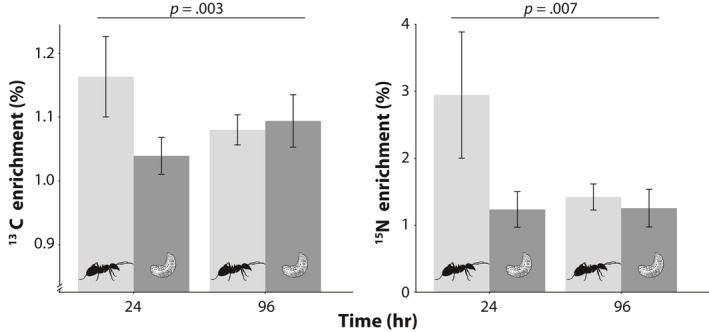
Atomic per cent excess in workers and larvae after a pulse of ^13^C and ^15^N isotopes. Workers became highly enriched rapidly (they collect the material), and then, food was transferred to larvae by 96 hr. While not statistically significant, the atomic enrichment of ^13^C trends toward being higher in larvae at 96 hr while there is no pattern of difference in ^15^N; this is opposite what was predicted. This assay was carried out with a single diet (1:4) in a subset of *Lasius neoniger* colonies. Variation is expressed as confidence intervals

There was a statistically significant effect of caste by sample time for atomic enrichment of both ^15^N and ^13^C (K‐W test, *χ* ^2^ > 12, *df* = 3, *p* < .005 for both ^15^N and ^13^C). It can be noticed that incoming nutrients are first ingested by workers, with worker atomic per cent of ^15^N and ^13^C peaking at 24 hr. The 24 hr workers had nearly double the atomic enrichment of ^13^C compared to all other treatments and had fivefold higher enrichment of ^15^N (Figure 7). Workers were statistically different (*p* < .05) from larvae at both 24‐ and 96‐hr collections for ^15^N, but the difference was not statistically significant in the 24–96 hr comparison for ^13^C. No other pairwise comparisons were statistically significant. We had predicted that we would see a movement of more nitrogen to larvae relative to carbon, but this was not evident in the data. However, if anything the opposite appears more likely. Larval atomic per cent ^15^N and ^13^C remained constant over both sample times. Larval C:N was much higher than that of adult workers, 7.24 ± 0.18 vs. 5.00 ± 0.11 (mean ± *SE*, *F*_1,39_ = 108.9, *p* < .001), likely due to the high lipid content of larvae. Thus, despite a high protein need relative to workers for growth, the carbohydrate needs of larvae were substantial.

### 3.4 Diet preference

**FIGURE 8:**
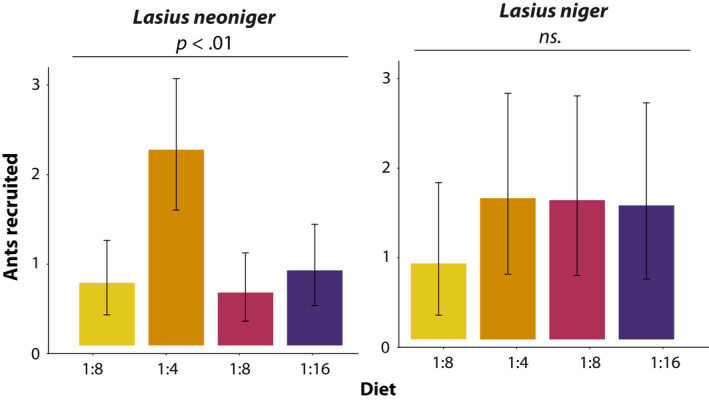
Field colonies of both *Lasius* species tended to avoid the diet with the highest protein content, and *L. neoniger* exhibited an overwhelming preference toward 1:4 diet. Means and confidence intervals were extracted from the mixed‐effects model using the effects library (Fox et al., 2019) and back‐transformed

About 20 baiting trials were conducted for each species in the field, but several time intervals and bait stations had to be discarded due to intrusion by other species or a lack of *Lasius* presence. *Lasius niger* clearly tended to avoid the diet with the highest protein content (1:2) but did not differentiate among the remaining diets (Figure 8), although pairwise comparisons revealed that 1:4 and 1:8 were significantly preferred over 1:2 (*p* < .05). On the other hand, *L. neoniger* exhibited an overwhelming preference for the 1:4 diet, with diet being highly significant in the mixed‐effects model (*χ* ^2^ = 52.01, *p* < .001). This trial was also replicated with two *L. neoniger* (ca. 2 years) laboratory colonies, and the result was qualitatively similar (data not shown).

In order to understand whether the preference for 1:4 was driven by the numerator (carbohydrates) or the denominator (protein) in the ratio, we offered new baits with both macronutrients to *L. neoniger* field colonies, yet in series where only one varied. We hypothesized that the preference for 1:4 was a composite of a combined preference for carbohydrates and proteins, especially because their response to the gradient in ratios was nonlinear. When carbohydrates were held constant, the ants had a strong preference for lower levels of protein; the number of ants presents with the least amount of protein was double that compared to the most protein. The intermediate level of protein received an intermediate level of visitation. The effect of diet in these trials was again highly statistically significant (*χ* ^2^ = 29.09, *p* < .001). When protein was held constant, the ants had an increasing preference for more carbohydrates, with the preference increasing with increasing levels of carbohydrates (*χ* ^2^ = 13.47, *p* = .001) (Figure S2). The laboratory results were consistent with the field data.

## 4 DISCUSSION

**FIGURE 9:**
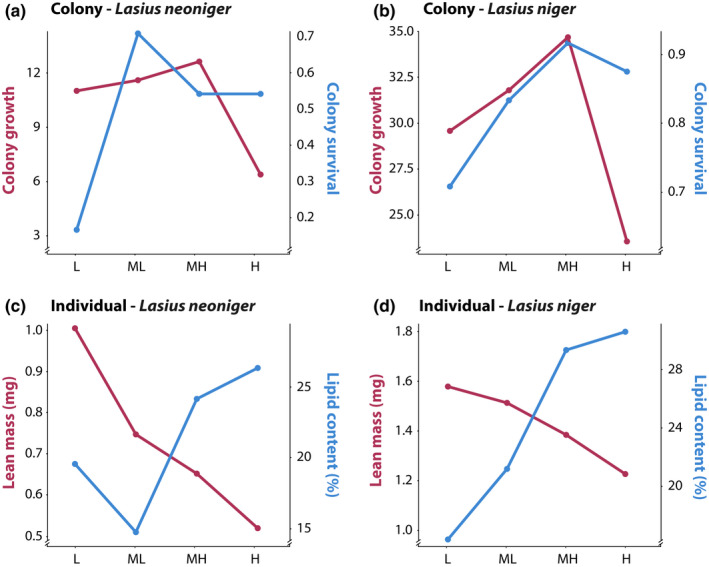
Untransformed values of colony survival and growth (a and b) and individual worker lean mass and lipid content (c and d). The lines intersect in all graphs, suggesting that both colony‐level growth and survival, as well as individual level lean and lipid mass, trade‐off as a function of the carbohydrate and protein content in the diet (x‐axis). In all cases, the lines intersect at the intermediate level (1:4 or 1:8) of P:C used in this experiment

We show evidence of nutrient trade‐offs for colony growth and survival, as well as for individual size and lipid storage. A visual schematic of nutrient trade‐offs is depicted in Figure 1, such that for a particular trait, there is a single optimal P:C. Trade‐offs exist when different traits have different optima, namely when the lines representing trait values over differing P:C values intersect. The lines for both colony (growth vs. survival) and individual traits (size vs. lipid content) intersect in our data (Figure 9). The area of intersection represents the optimal investment, assuming that each of the traits has equal fitness effect. For both individual and colony‐level traits, for both species, the intersection is at the level of the intermediate P:C used in this study. Interestingly, we may have detected the optimum P:C for both colony survival and growth; that is, intermediate P:C levels have the highest value of both survival and growth. On the other hand, the maximum values for the individual‐level traits of size and lipid content are at the extreme P:C levels used in this study—the largest workers were produced at the highest relative P and the fattest workers at the highest relative C. The existence of trade‐offs at the individual level suggests that colonies can manipulate the nutrients available to individuals within a nest in response to a changing external environment. For example, in response to environmental stress, colonies may modify the nutrients available to individuals within the nest, biasing provisioning toward carbohydrates over protein. At the colony level, a nutritionally mediated trade‐off between growth and survival implies that selection may optimize nutrient collection to benefit the life‐history trait most beneficial at a given time in the life cycle (including having variable strategies for nutrient collection).

### 4.1 Development of ant colonies

Individual workers and colonies (superorganisms) were smallest at the lowest P:C, and when less food was available, and yet there was no interaction between the two (Figures 4, 5, 6, 7 and 9). However, the high mortality exhibited by *L. neoniger* colonies under one of the experimental diets (L) is consistent with other studies that find either lesser stress resistance or nitrogen toxicity in high protein/low carbohydrate diets (Figure 4) (Cook, Eubanks, Gold, & Behmer, 2010; Dussutour & Simpson, 2009, 2012). We hypothesize that the high colony mortality was due to a decrease in lipid stores, as individual lipid content is a buffer for colony survival (Dussutour et al., 2016; Smith, 2007). The lower survival in *L. neoniger*, but not *L. niger*, with the highest protein (L) diet may be due to different overwintering conditions for each species. *Lasius neoniger* was overwintered at a higher temperature compared to *L. niger* (although the temperature transition was the same for both species), and thus, workers may have had higher metabolic rates and depleted greater amounts of their lipid reserves, leading to higher worker and colony mortality. In agreement with our data, lipid storage in other insects is increased with increased carbohydrate content of diet (Dussutour et al., 2016; Warbrick‐Smith, Behmer, Lee, Raubenheimer, & Simpson, 2006), and carbohydrates and lipids in the diet of ants may be largely equivalent (Rosumek, Brückner, Blüthgen, Menzel, & Heethoff, 2017). Note, the higher temperature used in *L. neoniger* was due to the concurrent overwintering of multiple, ecologically different, species.

Other studies have demonstrated that a change in the macronutrient ratios, toward a higher protein content, has toxic effects (Dussutour & Simpson, 2009, 2012; Harrison, Woods, & Roberts, 2012; Simpson & Raubenheimer, 2009). However, the P:C ratios in our study were all relatively high compared to studies documenting toxicity—increased protein content in our study was generally correlated with both increased worker number and worker size. A decrease in colony growth with even higher levels of protein was found in our preliminary study, which may have been due to a toxicity effect. Together, our preliminary data and the data presented in this paper suggest that optimal growth is achieved through a trade‐off between sufficient carbohydrates for lipid storage on the one hand, and enough protein for growth on the other (but less than will cause toxicity). In both *Lasius* species, this optimum appears to be at a P:C of 1:4 to 1:8. These results are consistent with previous studies using similar diets—mortality was lowest between 1:3 and 1:5 in *Rhytidoponera* sp., *L. niger* and *Solenipsis invicta* (Cook et al., 2010; Dussutour & Simpson, 2009, 2012).

Colony growth was nonlinear with respect to the amount of food provided. Colonies fed once per week did not grow any better than those fed twice weekly, suggesting that we found a maximum growth rate for colonies on the provided diets. Doubling food availability essentially saturated their ability to turn those nutrients into new ants. Colonies fed only once every 2 weeks, however, had decreased growth. Therefore, our levels of nutrient provisioning were appropriate to assess nutrient limitation as a function of both the amount and P:C ratio of provided diets.

### 4.2 Colony and individual phenotypes

Our study demonstrated that average ant size increased with increasing protein (relative to carbohydrates) and the total amount of diet provided. While we did not examine gene expression in this study, it has been established that caste determination in some social insects is regulated behaviorally and morphologically by nutrient‐sensing genes, including those involved in insulin and Tor signaling (Patel et al., 2007; Toth et al., 2007; Wheeler, Buck, & Evans, 2006; and reviewed in Chandra et al., 2018; Smith, Toth, Suarez, & Robinson, 2008; Toth & Robinson, 2007), and caste determination is largely explained by size variation (Trible & Kronauer, 2017). Thus, a universal feature of “organismality” is that size scales with the nutritional environment during development because the translation of nutrients into growth is achieved through conserved processes at the cellular level. If there existed a super‐superorganism, then we would also expect that its size, along with the size of its constituent parts, is nutritionally regulated; note that supercolonies, as seen in some ant species (Holway, Lach, Suarez, Tsutsui, & Case, 2002), do not likely fit the evolutionary definition of “organism.”

While worker size (such as head width or lean mass) increased with increasing protein in the diet, individual lipid content increased with increasing carbohydrates (Figure 6). As noted above, there was thus a trade‐off faced by organisms with regard to their nutritional choices for these two major macronutrients (Figure 9). Solitary insects, including caterpillars and last instar grasshoppers, tend to maximize growth with P:C near 1:1 (reviewed in Behmer, 2009), whereas flies performed better with a more carbohydrate bias (Lee et al., 2008; Young, Buckiewicz, & Long, 2018). Furthermore, it is clear that there are life‐history trade‐offs inherent in different P:C ratios, such as longevity (maximized with increasing decreasing protein) and egg laying (higher at increased protein) as shown for several model species (Clark, Zera, & Behmer, 2015; Gutiérrez, Fresch, Ott, Brockmeyer, & Scherber, 2020; Tatar & Carey, 1995).

While data are currently limited across insect taxa, we hypothesize that superorganism growth is maximized at a higher carbohydrate diet compared to most solitary insects. This prediction is premised on the majority of the standing biomass of a superorganism being nongrowing, nonreproducing workers. While larvae have a higher carbon to nitrogen (C:N) ratio than workers, this is largely due to their high lipid content. The colony's germline, reproductive gynes and males, have a higher nitrogen content (i.e., much lower C:N ratio) (Schmidt et al., 2012; Smith, Anderson, et al., 2008; Smith, Toth, et al., 2008; Smith & Suarez, 2010). A logical extension of this prediction is that social insect growth and development is less protein limited than solitary insects, and this may have been an inherent benefit to social living. This hypothesis is in line with how metabolic rate scales with body size in insect organisms and superorganisms. On a per mass basis, metabolism scales constantly across solitary and social insects (Hou, Kaspari, Vander Zanden, & Gillooly, 2010). Thus, eusociality and increased colony size are selectively advantageous with regard to increased metabolic efficiency.

### 4.3 Nutrient flows within colonies and colony‐level preferences

We predicted that larvae would receive incoming protein, disproportionately, compared to carbohydrates and that the opposite would be true for workers. Using stable isotope labeling of the most preferred and optimal diet of *L. neoniger*, fed as a pulse, we traced nitrogen (as a proxy for protein) and carbon (as a proxy for carbohydrates) through colonies over 4 days. As is necessarily the case, nutrients were collected by foraging workers, stored in their crops, and then transferred from workers to larvae via trophallaxis, but the signature of excess isotope enrichment in workers was gone in 4 days, suggesting that the diet was rapidly distributed (to other workers and larvae). Contrary to our expectation, after 4 days there were no differences between workers and larvae for either nutrient, and there, if anything (the result is marginally statistically significant), was the opposite pattern of nutrient transfer than predicted with larvae having slightly more of an excess of carbon than workers, but not nitrogen. Both workers and larvae at 4 days still showed atomic excess of labeled isotopes of both elements compared to colonies fed unenriched diets (i.e., they still had labeled nitrogen in/on their body). It is possible that proteins were being stored in workers until larvae were hungry—the timeframe of our study was insufficient to examine differences between developmental castes in diet assimilation. Additional studies using the basic isotope pulse strategy employed here, but with more time points and a higher resolution of sampling, will help disentangle some of the complex interactions within colonies that are difficult to infer from behavior, such as the relative distribution of nutrients to different subgroups within a nest, and how rapidly they are distributed and assimilated (Shik et al., 2018).

Workers are nongrowing and thus feed primarily on carbohydrates to fuel their metabolism. As discussed above, we show that colony growth is maximized at intermediate levels of protein in the diet. When given choices of the same diets used to assay colony growth, colony preference was not in alignment with the optimum for colony growth in *L. niger*—higher protein diets increased growth, but higher carbohydrate diets were preferred. In *L. neoniger*, on the other hand, colony preference was uncannily aligned with optimal colony growth (data were consistent between field and laboratory colonies). Further work done only with *L. neoniger* found that preference for protein and carbohydrates in the diet is a result of clear preferences for each of the varying macronutrients, and thus, the ants are judging the ratio. When each protein and carbohydrates were manipulated individually, the ants had a clear preference for increasing carbohydrates and for decreasing amounts of protein. What caused a decrease in preference at the higher levels of carbohydrates in the first preference experiment on *L. neoniger* is unclear, but the effect persisted across multiple preparations of the diets and was consistent in the laboratory and field (Figure 8, Figure S2).

It is difficult to draw generalizable conclusions from a single (one‐season) diet/bait preference assay, and it is also difficult to judge optimality in diet from growth only under laboratory conditions. That being said, increased carbohydrate preference by foragers may be an adaptive strategy because this prioritizes survival over growth, and ant colonies tend to be long‐lived. For example, when starved, colonies will prioritize investment in growth over investment in reproduction (Smith, 2007), presumably because they will have future attempts at reproduction should they survive the current period of low resource levels. Preference/foraging, though, may not be optimal due to many types of constraints (Pyke, 1984), and studies on some ants have failed to tightly link forager preference with productivity/fitness (Seal & Tschinkel, 2007). Foragers are responsive to the presence of larvae in the nest and adjust the collection of resources to more protein in their presence (Cassill & Tschinkel, 1999; Dussutour & Simpson, 2009). Therefore, it is not as though overall colony preference, as expressed through foragers, is not regulated by feedback. Furthermore, ants are capable of filtering nutrients once foragers return to the colony. Although in nonsocial organisms this mechanism would be composed mainly by selective absorption of nutrients and excretion of excess, ants use the larvae as a protein stomach and the nutrients are distributed across nest members (Cassill, Butler, Vinson, & Wheeler, 2005; Greenwald, Baltiansky, & Feinerman, 2018; Greenwald, Eckmann, & Feinerman, 2019; Greenwald, Segre, & Feinerman, 2015; Sorenşen, Busch, & Vinson, 1985; Wilson, 1976), or to stores/trash enriched with protein (Dussutour & Simpson, 2009).

## 5 CONCLUSION

We have shown that macronutrient effects scale up to limit the size of both organisms and superorganisms. The selective benefits of size, such as task efficiency, longevity, and metabolic efficiency, also trade‐off with physiological resource storage. These factors are predicted to select for tuning of dietary preferences, and we found evidence of dietary preference—and ants appear to make decisions on the diet by assessing multiple ingredients independently. The mechanisms by which superorganisms integrate nutritional information and translate nutrition into growth decisions, including feedbacks, are only beginning to be elucidated.

## CONFLICT OF INTEREST

The authors declare that they have no competing interests.

## AUTHOR CONTRIBUTIONS

**Yeisson Gutiérrez:** Conceptualization (equal); Data curation (lead); Formal analysis (lead); Investigation (lead); Methodology (equal); Visualization (lead); Writing‐original draft (supporting); Writing‐review & editing (equal). **Tung Phung:** Investigation (equal). **Harald Mumma:** Investigation (equal). **Arden Ambrose‐Winters:** Investigation (equal). **Christoph Scherber:** Funding acquisition (equal); Resources (equal); Supervision (equal); Writing‐review & editing (equal). **Chris Smith:** Conceptualization (equal); Formal analysis (equal); Funding acquisition (equal); Investigation (equal); Methodology (equal); Project administration (lead); Supervision (equal); Visualization (equal); Writing‐original draft (lead); Writing‐review & editing (lead).

## ETHICAL APPROVAL

All applicable national guidelines for the care and use of animals were followed. Permission for *L. niger* collection was granted by the city of Münster (67‐20‐0032, 2017).

## Supporting information

Supplementary Material

## Data Availability

All raw data and associated analysis code (in R) are publicly available in the Dryad repository. The following is a reviewer URL: https://datadryad.org/stash/share/79aIqBm0qQZhZ0fXCnlZEAnzV5iF7lHZy3UOssit0jU.
