# Supplementary material for: Growth and survival of the superorganism: Ant colony macronutrient intake and investment
Source: Ecol Evol. 2020 Jul 7;10(14):7901–15. doi: 10.1002/ece3.6520 (PMC7391535; doi:10.1002/ece3.6520)

***Pilot study***

In a preliminary experiment, twenty-four *L. neoniger* colonies were monitored over six months; colonies were distributed among three treatments varying in the protein to carbohydrate content of their diet. Colony growth (number of workers) increased near linearly with increasing parts of carbohydrates, where colonies fed a diet with a P:C ratio of 1:1 produced fewer workers than those fed a diet of 1:2, and those fed a diet of 1:3 produced the greatest number of workers. The experiment also documented that a weekly provision of ~125 µl of artificial diet (see below) was sufficient to maintain colony growth and survival.  These pilot data informed our choice of the quantity of diet to provision and the P:C range.

**Figure S1.** Results of pilot experiment. 1, 2, 3 represent the amount of carbohydrate relative to a constant amount of protein (thus these are 1:1, 1:2, and 1:3 P:C). Letters represent differences in means at P < 0.05 in a Tukey HSD test.

**Table S1.** Diets’ recipes used for the experiments. Values differ because of variation of nutritional composition of the ingredients. All ingredients are given in grams.

| Species | Diet (P:C) | EggPowder | Whey | Casein | Sugar |
| --- | --- | --- | --- | --- | --- |
| *Lasius niger* | 1:2 | 9.128 | 10.486 | 11.312 | 39.074 |
|  | 1:4 | 9.128 | 5.439 | 5.866 | 49.560 |
|  | 1:8 | 9.128 | 2.069 | 2.232 | 56.567 |
|  | 1:16 | 9.128 | 0 | 0 | 60.872 |
| *Lasius neoniger* | 1:1.7 | 9.391 | 12.255 | 11.989 | 38.364 |
|  | 1:3 | 9.391 | 7.599 | 7.434 | 47.576 |
|  | 1:5 | 9.391 | 4.318 | 4.224 | 54.067 |
|  | 1:14.5 | 9.391 | 0 | 0 | 62.609 |

The previously detailed ingredients sum 72g for each diet, those are mixed with 2g of Insect vitamins (Vandersant, MP Biomedicals, Germany) and 0.3g of preservative (Methylparaben, Dephyte, Germany) in a boiled solution of 300ml of water with 4g of agar.

Agar is a non-digestible polymer of galactose extracted from seaweed. Therefore, is not included in the calculation of total carbohydrates.

**Table S2.** Approximate nutritional composition of ingredients used for preparing the diets

|  | **Egg Powder** | **Whey** | **Casein** | **Sugar/Sucrose** |
| --- | --- | --- | --- | --- |
| Carbs(g) | 0.17 | 0.04 | 0.04 | 1 |
| Prot(g) | 0.45 | 0.82 | 0.76 | 0 |
| Fat(g) | 0.38 | 0.075 | 0.015 | 0 |
| Cal | 5.9 | 4.12 | 3.56 | 3.75 |
| total (g) | 1 | 1 | 1 | 1 |

**Stable isotope pulse diet:** We modified the diet above for the stable isotope pulse experiment. Ammonium nitrate and glucose were added to the standard *L. neoniger* 1:4 diet at the same ratio as the normal diet (glucose for parts C and ammonium nitrate for parts P, above). The glucose and ammonium nitrate were unlabeled for control colonies and labeled for pulse colonies. Labeled: Ammonium nitrate-15N, EB0056; unlabeled: Ammonium Nitrate SHBJ1050, Sigma-Aldrich, USA. Labeled: D-Glucose-1-13C, MBBC0227V; unlabeled: D-(+)-Glucose, SLBJ0583V, Sigma-Aldrich, USA).

**Table S3.** Two-way ANOVA results for overall colony growth. df= degrees of freedom.

|  | ***Lasius neoniger*** | | |  | ***Lasius niger*** | | |
| --- | --- | --- | --- | --- | --- | --- | --- |
|  | **df** | ***F*** | ***P*** |  | **df** | ***F*** | ***P*** |
| Diet (P:C) | 3 | 0.5970 | 0.6213 |  | 3 | 4.364 | 0.00718 |
| Frequency | 2 | 3.8860 | 0.0296 |  | 2 | 14.348 | <0.001 |
| Diet * Frequency | 5 | 0.3540 | 0.8766 |  | 6 | 0.517 | 0.79341 |

**Table S4.** Linear and generalized linear models results for workers phenotypes. df= degrees of freedom

|  | ***Lasius neoniger*** | | | | | | | | | | | | |
| --- | --- | --- | --- | --- | --- | --- | --- | --- | --- | --- | --- | --- | --- |
|  |  |  | **Head width** | |  | **Dry mass** | |  | **Lean mass** | |  | **Lipid content** | |
|  | **df** |  | ***χ*^2^** | ***P*** |  | **F** | ***P*** |  | **F** | ***P*** |  | **F** | ***P*** |
| Diet | 3 |  | 2.2994 | 0.5126 |  | 3.2870 | 0.0352 |  | 6.2123 | 0.0018 |  | 4.0154 | 0.0170 |
| Frequency | 2 |  | 8.8529 | 0.0120 |  | 3.6130 | 0.0402 |  | 0.9630 | 0.3940 |  | 2.2520 | 0.1239 |
| Diet * Frequency | 5 |  | 7.9509 | 0.1590 |  | 0.7300 | 0.6069 |  | 0.8030 | 0.5570 |  | 1.6950 | 0.1680 |
|  |  |  |  |  |  |  |  |  |  |  |  |  |  |
|  | ***Lasius niger*** | | | | | | | | | | | | |
|  |  |  | **Head width** | |  | **Dry mass** | |  | **Lean mass** | |  | **Lipid content** | |
|  | **df** |  | ***χ*^2^** | ***P*** |  | **F** | ***P*** |  | **F** | ***P*** |  | **F** | ***P*** |
| Diet | 3 |  | 87.6520 | <0.001 |  | 3.1683 | 0.0300 |  | 14.7482 | <0.001 |  | 29.7860 | <0.001 |
| Frequency | 2 |  | 89.6230 | <0.001 |  | 22.0120 | <0.001 |  | 8.9420 | <0.001 |  | 11.8600 | <0.001 |
| Diet * Frequency | 6 |  | 76.4720 | <0.001 |  | 0.9550 | 0.4626 |  | 1.2690 | 0.2835 |  | 1.3500 | 0.2480 |

**Figure S2.** Diet preference (recruitment) in field and laboratory colonies of *Lasius neoniger*


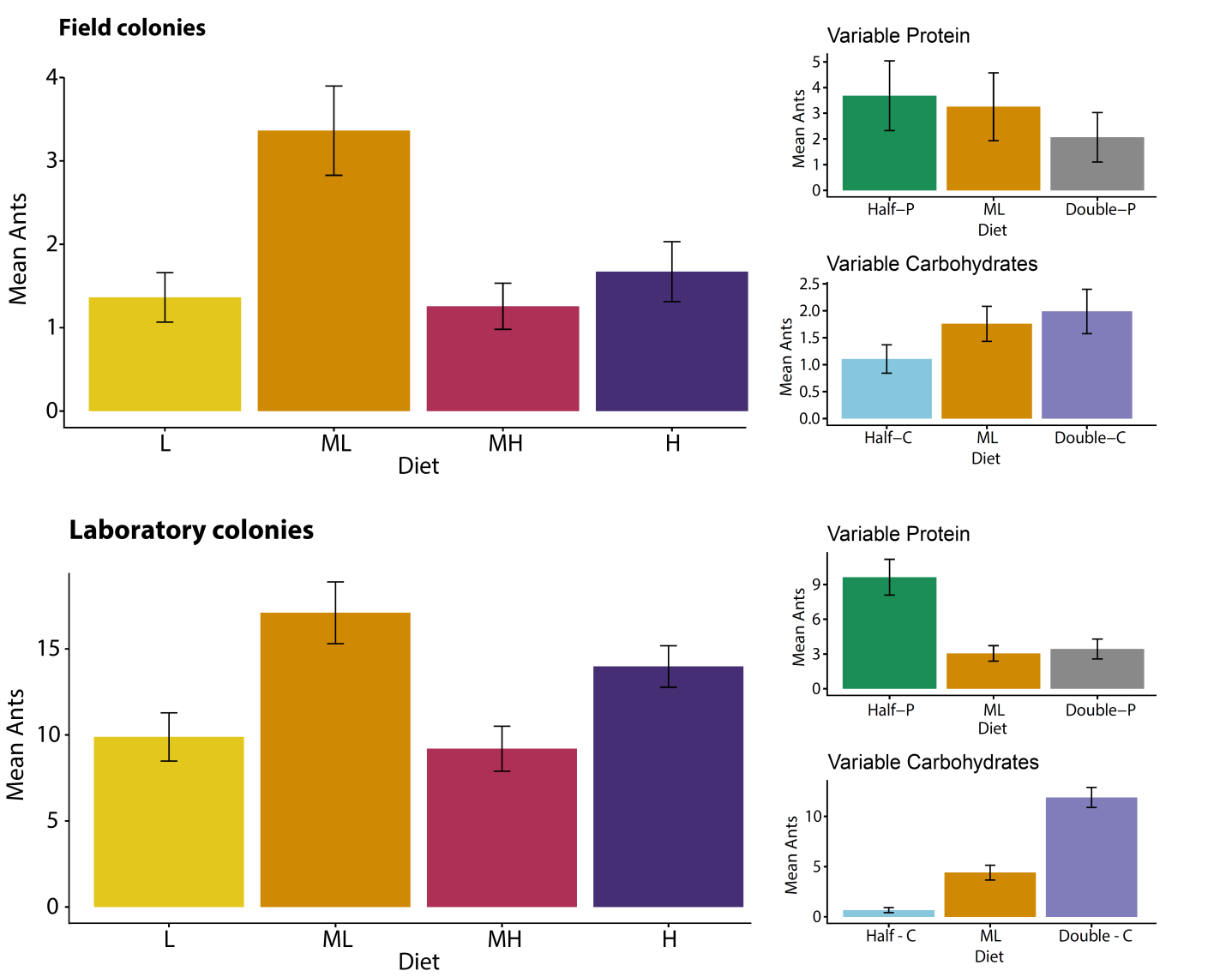

Supplement: Supplementary file 1 — Supplementary Material [file ECE3-10-7901-s001.docx]
